# Association of duration and etiology with the effect of the artificial liver support system in pediatric acute liver failure

**DOI:** 10.3389/fped.2022.951443

**Published:** 2022-10-20

**Authors:** Chun-Feng Yang, Jing-Wei Liu, Lin-Mei Jin, Yu-Mei Li

**Affiliations:** Department of Pediatric Intensive Care Unit, First Hospital of Jilin University, Changchun, China

**Keywords:** pediatric acute liver failure, artificial liver support system, extracorporeal purification, risk factors, plasma exchange

## Abstract

**Background:**

We aimed to assess the efficacy of the artificial liver support system (ALSS) in pediatric acute liver failure (PALF) patients and to examine the risk factors associated with the effect of ALSS. Similar data are limited in PALF.

**Methods:**

All patients diagnosed with PALF who received ALSS from June 2011 to June 2021 in the pediatric intensive care unit of the First Hospital of Jilin University were included in this retrospective cohort analysis. The effect of ALSS was measured using difference tests before and after treatments. The risk factors associated with the effect of ALSS were evaluated according to whether the total bilirubin (TBIL) and serum ammonia decreased after ALSS (TBIL-unresponsive group vs. TBIL-responsive group, serum ammonia-unresponsive group vs. serum ammonia-responsive group).

**Results:**

Thirty-nine patients who received ALSS during the study period were eligible for inclusion. The most common cause of PALF was undetermined causes (*n* = 14, 35.9%) followed by infection (*n* = 11, 28.2%). Four patients received pediatric liver transplantation. The overall survival rate was 76.9% (30/39). Fifteen (38.4%) patients received only one modality, whereas 61.6% patients received hybrid treatments. The most commonly used modality of ALSS was plasma exchange combined with continuous renal replacement therapy (*n* = 14, 35.9%). Alanine aminotransferase, TBIL, the international normalized ratio, and serum ammonia were significantly decreased after ALSS (*P* < 0.001). Compared with other causes, more patients with infection and toxication were observed in the TBIL-unresponsive group. A longer ALSS duration was significantly related to blood ammonia reduction.

**Conclusions:**

ALSS can effectively reduce serum alanine aminotransferase, TBIL, international normalized ratio, and serum ammonia and may reduce mortality. The reduction in TBIL levels after ALSS is dependent on etiology. A longer ALSS duration was associated with blood ammonia reduction. Prospective multicenter studies are needed for further validation.

## Introduction

Pediatric acute liver failure (PALF) is a rare but devastating disease that occurs in previously healthy children and rapidly leads to multiple organ dysfunction syndrome (MODS) and death (1). The exact pathophysiology of MODS in PALF remains elusive, but toxins and metabolites leading to the systemic inflammatory response play an important role in hepatocyte damage (2). Pediatric liver transplantation is considered to be the most effective therapy but is limited by the lack of donors. In addition, the recipients also suffer from transplant-related morbidity and mortality. Hence, spontaneous recovery with the native liver remains the optimal outcome in PALF.

The artificial liver support system (ALSS) providing conditions for spontaneous regeneration or bridging to liver transplantation plays an essential role in the management of PALF. ALSS is a type of extracorporeal support system including plasma exchange (PE), continuous renal replacement therapy (CRRT), double plasma molecular adsorbent system (DPMAS), and single pass albumin dialysis, which can be used alone or in hybrid methods. Many studies in critically ill adults have demonstrated the biochemical efficacy and survival benefit of various modalities of ALSS in acute liver failure or acute-on-chronic liver failure (3–5). Among these, PE is the most commonly used modality in China. In recent guidelines proposed by the American Society of Apheresis, high-volume PE was accepted as a strong grade 1A/III recommendation for acute liver failure, and nonhigh volume PE was regarded as a weak grade 2B/III recommendation due to a lack of reliable evidence (6, 7). In PALF, data on extracorporeal systems are scarce (8). Therefore, the lack of reliable evidence makes it challenging to judge the efficacy of ALSS in pediatrics. Extrapolation of evidence from adult data is ambiguous because PALF differs from adult liver failure based on etiologies, pathophysiology, comorbidities, treatments and prognosis (9). To date, few studies have explored the factors that influence ALSS in PALF.

The first endpoint for this study was the efficiency of ALSS in PALF. The secondary endpoint was variations in relation to the effect of ALSS. We hope this study will help predict PALF clinical outcomes and the need for liver transplantation.

## Materials and methods

### Patients and setting

This single-center retrospective observational study was conducted in a tertiary referral 20-bed pediatric intensive care unit (PICU) over a 10-year period in northeast China. We reviewed all hospitalized pediatric patients diagnosed with PALF from June 2011 to June 2021 in the PICU of the First Hospital of Jilin University. The children were eligible for inclusion in the study if they were diagnosed within 24 h and underwent at least one modality of ALSS, including CRRT, PE, and DPMAS. PALF was defined according to the Pediatric Acute Liver Failure Study Group criteria (10). The exclusion criteria were (a) a PICU stay less than 48 h and (b) a Glasgow Coma Scale score of 3 at admission. The study protocol was approved by the Ethics Committee of the First Hospital of Jilin University. Because the study was retrospective, our committee waived the requirement for patient consent.

### Data collection

Patient records in the institutional electronic health record database were manually extracted and reviewed by two investigators. Data elements included age, sex, pediatric risk of mortality III (PRISM III) scores, interval time between onset and PICU admission, primary diagnoses, hepatic encephalopathy (HE) (West Haven criteria) at admission, etiologies, time to initiate ALSS from PICU admission, alanine aminotransferase (ALT), total bilirubin (TBIL), international normalized ratio (INR) and serum ammonia, blood glucose before and after ALSS, various ALSS modalities, complications, the need for mechanical ventilation and vasoactive agents, ventilation duration, length of PICU stay, length of hospital stay, and outcomes.

Hypoglycemia was noted when blood glucose was less than 50 mg/dl (2.8 mmol/L), and hyperglycemia was diagnosed if a random blood glucose was greater than 200 mg/dl (>11.1 mmol/L).

### Group

Common biochemical markers assessed in liver failure include ALT, TBIL, INR and serum ammonia. In liver failure, enzyme-biliary separation may occur, so the level of ALT does not necessarily reflect disease severity. INR is greatly affected by plasma or the human prothrombin complex. Finally, we chose the clearance of TBIL and serum ammonia to represent the efficiency of ALSS. Based on whether TBIL decreased after ALSS treatment, the patients were divided into a TBIL-responsive group and a TBIL-unresponsive group. A patient was included in the TBIL-responsive group when the first TBIL value after ALSS was lower than the TBIL value obtain before ALSS. A patient was included in the TBIL-unresponsive group when the first TBIL value after ALSS was similar to or greater than the TBIL value obtained before ALSS. Variables were compared between the two groups to explore the risk factors influencing the effect of ALSS on TBIL reduction. During data analysis, patients with normal TBIL before and after ALSS were excluded. The same process was used to assess the risk factors associated with the effect of ALSS on serum ammonia.

### Procedure

All patients received disease-specific therapies and comprehensive medical treatment. Fresh frozen plasma (5~10 ml/kg, once daily, not transfused on the day of PE) and human prothrombin complex (including clotting factors II, VII, IX, X, 10~20 IU/kg, twice a day) were used to improve coagulopathy. Platelets were not routinely offered in the absence of bleeding. Red blood cells (5~10 ml/kg) were infused when the patient exhibited moderately or worse anemia with hemorrhage or hemodynamic instability. The other management strategies included energy and vitamin supplementation, infection control, blood glucose and blood pressure regulation, prevention of gastrointestinal bleeding and HE.

We investigated the precise etiology of PALF at admission. The diagnosis of drug-induced PALF was based on a history of exposure to the drug, clinical presentation and biochemical pattern with Roussel Uclaf Causality Assessment Method scores greater than or equal to 6. The diagnosis of mushroom poisoning was based on gastrointestinal symptoms after ingestion of wild mushrooms and the absence of other possible causes. Screening for viruses using nucleic acid testing and serology was undertaken to confirm infection. For nonviral infections, we focused on bacterial and fungal infections. Because we were not located in the endemic area, we did not routinely screen for parasites and other rare epidemic pathogens. The assessment of metabolic diseases and mitochondrial disorders included positive family history, recurrent episodes of metabolic decompensation and multiorgan involvement, plasma or urine amino acid analysis, organic acid analysis and genetic testing. The diagnosis of autoimmune-PALF was based on raised transaminases, positive autoantibodies, high immunoglobulin G, and exclusion of other diseases (11).

**Figure 1:**
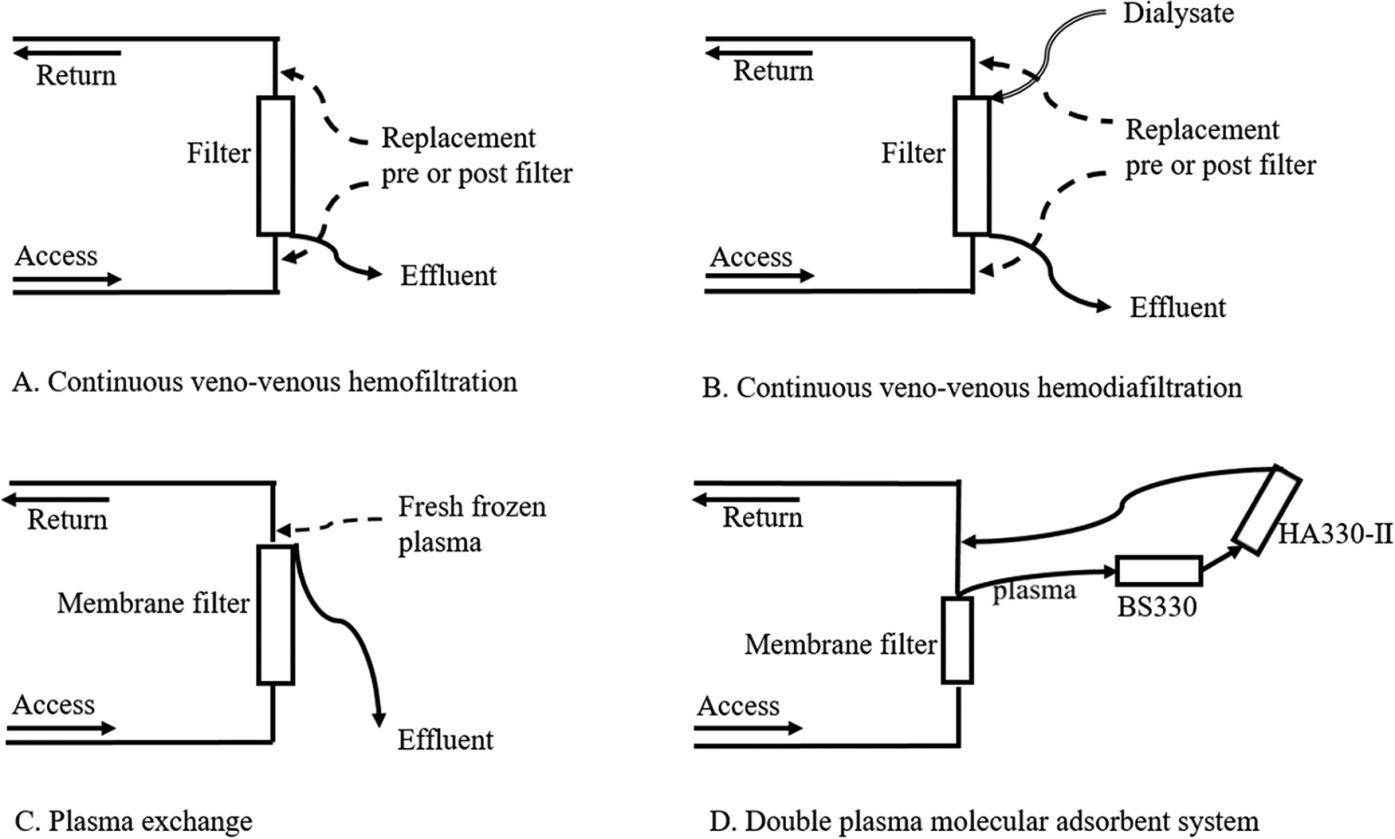
Schematic diagrams of different modalities of artificial liver support systems.

The decision to perform ALSS was made by the pediatric intensivist when the diagnosis of PALF was confirmed. After the parents' consent was obtained, preparation for ALSS was initiated. To date, the use of ALSS in PALF has not been protocolized. The indications, modality, anticoagulation, treatment dose, and timings for initiation and suspension were in accordance with the local protocols. There are three basic patterns of ALSS in our department, including CRRT, PE and DPMAS, and their schematic diagrams are shown in Figure 1.

In general, PE was the preferred mode for all patients with PALF. PE was performed using a continuous renal replacement machine (multiFiltrate, Fresenius Medical Care AG/Co. KGaA, Germany), a membrane filter (Plasma Flux P1/P2 dry, Fresenius Medical Care AG/Co. KGaA, Germany) and extracorporeal circuits (MPS Substituate System multiFiltrate and multiFiltrate Cassette, Fresenius Medical Care AG/Co. KGaA, Germany). It replaced 50 ml/kg of fresh frozen plasma per treatment course for 2 h. The blood flow rate was 3~5 ml/kg/min with a maximum of 150 ml/min. No anticoagulants were used. Ideally, PE is performed as soon as the patients met the diagnostic criteria of PALF followed by once daily until coagulation and liver function improved or death. However, in practice, the timing and frequency of PE are limited by the availability of plasma, and the actual interval is 1 to 2 days.

DPMAS was selected when PE was not performed in a timely manner and bilirubin levels were significantly elevated (TBIL > 200 μmol/L). DPMAS was applied using a continuous renal replacement machine (multiFiltrate, Fresenius Medical Care AG/Co. KGaA, Germany) and a hemoperfusion machine (JF—800 A, Zhuhai Health Sails Biotechnology Co., Ltd., Zhuhai, China). The membrane filters and extracorporeal circuits used in DPMAS were the same as those used in PE. DPMAS combined two types of absorbents, including absorption resin (HA330-II, Zhuhai Health Sails Biotechnology Co., Ltd., Zhuhai, China) and ion exchange resin (BS330, Zhuhai Health Sails Biotechnology Co., Ltd., Zhuhai, China). Each session of the DPMAS was 2 h. The blood flow rate was 3~5 ml/kg/min with a maximum of 150 ml/min. No anticoagulants were used.

Indications for CRRT in PALF were (1) elevated ammonia accompanied by grade III or IV HE, (2) MODS, (3) acute kidney injury (AKI) and (4) fluid overload. CRRT was performed using a continuous renal replacement machine (multiFiltrate, Fresenius Medical Care AG/Co. KGaA, Germany), an extracorporeal circuit (multiFiltrate Cassette, Fresenius Medical Care AG/Co. KGaA, Germany), and a membrane filter (Ultraflux AV paed/AV400S/AV600S, Fresenius Medical Care AG/Co. KGaA, Germany). In patients weighing more than 10 kg, continuous veno-venous hemodiafiltration was performed with a dialysate flow rate of 20~30 ml/kg/h and a predilution filtration rate of 30~50 ml/kg/h. In patients weighing less than 10 kg, continuous veno-venous hemofiltration was performed with a predilution filtration rate of 30~50 ml/kg/h. The blood flow rate was 3–5 ml/kg/min. Anticoagulation was achieved by regional citrate infusion. Target postfilter ionized calcium concentrations were 0.4~0.6 mmol/L. Dialysate and replacement fluids were prepared by PICU nurses strictly according to a standardized protocol.

**Figure 2:**
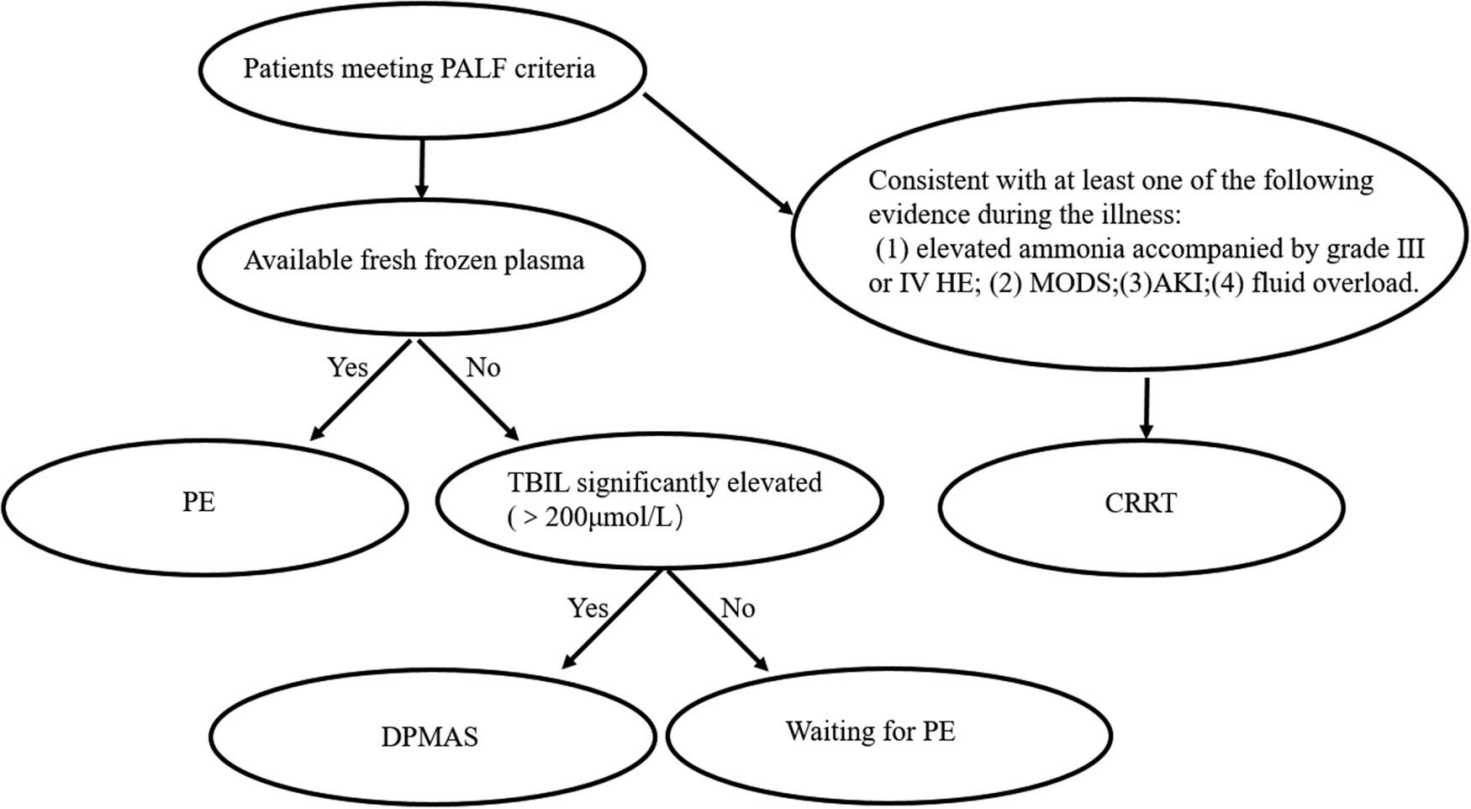
A general procedure for selecting ALSS modalities for patients with PALF.

A general procedure for selecting ALSS modalities for patients with PALF in our institution is shown in Figure 2.

ALSS was performed until coagulation and liver function improved or the outcome event (liver transplantation or death) occurred. All the extracorporeal treatments were generally well tolerated.

### Statistical analysis

Statistical analysis was performed using SPSS 24.0 software (SPSS Inc., Chicago, Illinois). The hypothesis of a normal distribution was tested with the Kolmogorov‒Smirnov test and histograms. Data are presented as the mean and standard deviation (SD) when normally distributed and otherwise as the median and interquartile range (IQR). Categorical variables were expressed as numbers and percentages. For the data with a nonnormal distribution, the Wilcoxon rank sum test for paired sample comparison was used to compare biochemical parameters before and after ALSS, and the independent sample Mann‒Whitney U rank sum test was used to compare variables between groups. Categorical variables were calculated using Fisher's exact test given that the total sample number was less than 40. After identifying factors that may be related to the efficiency of ALSS through univariate analysis, we next performed univariate or multivariate regression and Spearman rank correlation analysis with the outcome. *P *< 0.05 was considered statistically significant.

## Results

### Patient characteristics

**Figure 3:**
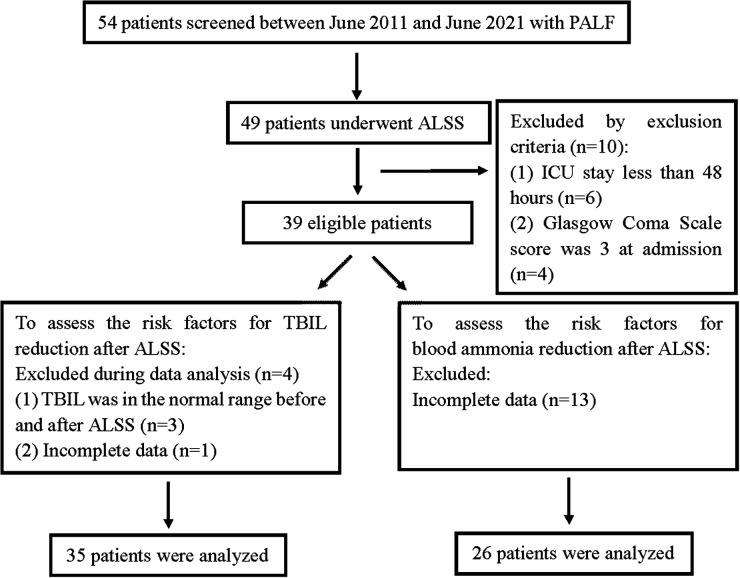
Flow chart of study participants.

From June 2011 to June 2021, a total of 54 patients were diagnosed with PALF in the PICU of our hospital. Thirty-nine patients who received ALSS were eligible (see the patient flowchart in Figure 3). The median age was 7 years old. The most common cause of PALF was indeterminate causes (*n* = 14, 35.9%) followed by infection (*n* = 11, 28.2%), genetic metabolic diseases (*n* = 9, 23.1%), and toxication (*n* = 5, 12.8%). The PRISM III score was 11.7 ± 6.4. Patients with HE at admission accounted for 30.8% of all cases. More than three failed organs were noted in twenty (51.2%) patients, and AKI was present in 12.8% of the patients. Approximately half of the patients were mechanically ventilated (*n* = 22, 56.4%) and required vasopressor support (*n* = 20, 51.2%). The median PICU length of stay and hospital stay were 13 days and 17 days, respectively. Four patients received pediatric liver transplantation. The overall survival rate was 76.9% (30/39). Patient characteristics are shown in Table 1.

**Table 1:** Baseline characteristics of the study population (*n* = 39).

Variable	Outcome
Age (year), median (IQR)	7 (1, 10)
Male (*n*, %)	16 (41%)
PRISM III, mean (SD)	11.7 (6.4)
HE at admission (*n*, %)	12 (30.8%)
Onset to PICU (d), median (IQR)	6 (3, 9)
Etiology (*n*, %)	
Infection	11 (28.2%)
Toxication	5 (12.8%)
Indeterminate causes	14 (35.9%)
Metabolic diseases	9 (23.1%)
ALSS start time (h), median (IQR)	26 (15, 48)
ALSS modalities (*n*, %)	
PE only	7 (17.9%)
DPMAS only	1 (2.5%)
CRRT only	7 (17.9%)
PE + CRRT	14 (35.9%)
PE + DPMAS	6 (15.4%)
PE + DPMAS + CRRT	4 (10.2%)
Complications	
HE (*n*, %)	26 (66.7%)
AKI (*n*, %)	5 (12.8%)
MODS no. ≥3 (*n*, %)	20 (51.2%)
Ventilation (*n*, %)	22 (56.4%)
Ventilation duration (d), median (IQR)	4.5 (3, 10.5)
Vasoactive agents (*n*, %)	20 (51.2%)
ICU stay (d), median (IQR)	13 (6, 17)
Hospital stay (d), median (IQR)	17 (10, 26)
Survivors (*n*, %)	30 (76.9%)

PRISM, pediatric risk of mortality III; HE, hepatic encephalopathy; ALSS, artificial liver support system; PE, plasma exchange; DPMAS, double plasma molecular adsorption system; CRRT, continuous renal replacement therapy; AKI, acute kidney injury; MODS, multiple organ dysfunction syndrome.

### Effects of ALSS

Fifteen (38.4%) patients received only one modality (PE, DPMAS or CRRT only), whereas 61.6% patients received the hybrid artificial liver support modality. The most commonly used modality of ALSS was PE combined with CRRT (*n* = 14, 35.9%) (Table 1). Thirty-one patients (79.5%) received PE, and eleven patients (28.2%) underwent DPMAS. Both had a median frequency of 2. The CRRT duration was 3 (2, 5) days in twenty-five (64.1%) patients who underwent CRRT.

In the overall population (*n* = 39), ALT before ALSS was significantly decreased compared to that after the procedure (before: 718 (80, 1666) U/l vs. after: 189 (79, 595) U/l, *P* < 0.001), and a significant decline in TBIL was observed (before: 134 (54, 256) μmol/l vs. after: 84 (39, 175) μmol/l, *P *= 0.001). The significant improvement in coagulation was characterized by a reduction in the INR (before: 1.98 (1.5, 2.7) vs. after: 1.55 (1.3, 2), *P* < 0.001). A significant reduction in serum ammonia was noted during the observation period (before: 113 (81, 181) μmol/l vs. after: 89 (67, 129) μmol/L, *P *= 0.049). With respect to glucose levels, six cases (15.3%) experienced transient hypoglycemia before ALSS. Among them, only one fasting patient required continuous infusion of parenteral nutrition (glucose infusion rate 3 mg/kg.min) to maintain blood glucose, whereas hypoglycemia was corrected after an intravenous 2 ml/kg bolus of 10% dextrose in the remaining cases. Blood glucose increased significantly after ALSS (before: 5.4 (4.5, 6.4) mmol/L vs. after: 7.1 (5.4, 8.9) mmol/l, *P* = 0.002). After the treatments, all the survivors did not need continuous glucose infusion to maintain blood glucose.

### Risk factors on the efficiency of ALSS

**Figure 4:**
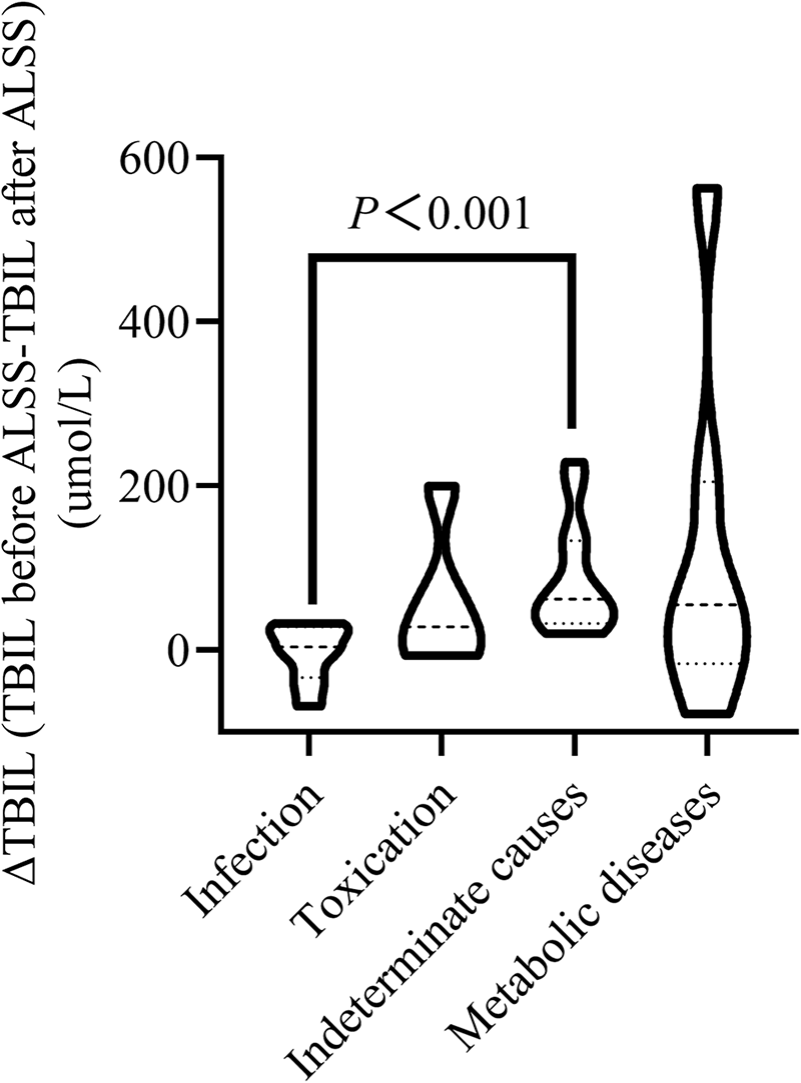
The change in the total bilirubin level after ALSS based on different etiologies.

To examine factors influencing TBIL reduction after ALSS, the patients in the study cohort were divided into a TBIL-responsive group (TBIL _before ALSS _> TBIL _after ALSS_, *n* = 24) and a TBIL-unresponsive group (TBIL _before ALSS_ ≈ or < TBIL _after ALSS_, *n* = 11) according to whether TBIL was reduced. Among the multiple variables compared between the two groups, etiology was the only factor that was statistically significant, so we did not perform a multifactor analysis (Table 2). Compared with other causes, more patients with infection and toxication were noted in the TBIL-unresponsive group (*P *= 0.027), and their reduction in TBIL was poor (Table 2, Figure 4).

**Table 2:** Univariate analysis between the TBIL-responsive group and TBIL-unresponsive group.

	All (*n* = 35)	TBIL-responsive group (*n* = 24)	TBIL-unresponsive group (*n* = 11)	*P* value
Age (year), median (IQR)	7 (1.5, 10)	8 (1.5, 12)	6.5 (0.7, 9.2)	0.158^b^
Male (*n*, %)	15 (42.8%)	8 (33.3%)	7 (63.6%)	0.144^a^
PRISM III, median (IQR)	13 (6, 18)	13 (5, 17.5)	10.5 (7.7, 18.5)	0.725^b^
HE at admission (*n*, %)	11 (31.4%)	8 (33.3%)	3 (27.3%)	1^a^
Onset to PICU (d), median (IQR)	6 (3, 9)	7 (4.5, 10)	7 (2.8, 8)	0.545^b^
Etiology (*n*, %)				
Infection/toxication	15 (42.8%)	7 (29.2%)	8 (72.7%)	0.027^a^
Other etiology	20 (57.2%)	17 (70.8%)	3 (27.3%)	
ALSS start time (h), median (IQR)	26 (15, 48)	25 (17.2, 48)	26 (6, 28.8)	0.46^b^
ALSS modalities (*n*, %)				
PE only	6 (17.1%)	4 (16.7%)	2 (18.2%)	0.373^a^
DPMAS only	1 (2.9%)	1 (4.2%)	0	
CRRT only	7 (20%)	2 (8.3%)	4 (36.4%)	
PE + CRRT	12 (34.3%)	9 (37.5%)	4 (36.4%)	
PE + DPMAS	4 (11.4%)	4 (16.7%)	1 (9.1%)	
PE + DPMAS + CRRT	4 (11.4%)	4 (16.7%)	0	
Complications				
HE (*n*, %)	23 (65.7%)	16 (66.7%)	7 (63.6%)	1^a^
AKI (*n*, %)	4 (11.4%)	3 (12.5%)	1 (9.1%)	1^a^
MODS no. ≥ 3 (*n*, %)	19 (54.3%)	13 (54.2%)	6 (54.5%)	1^a^
Ventilation (*n*, %)	20 (57.1%)	13 (52%)	7 (63.6%)	0.721^a^
Ventilation duration (d), median (IQR)	4.5 (3, 10.5)	4 (2.5, 7.5)	8 (3, 15)	0.23^b^
Vasoactive agents (*n*, %)	17 (48.6%)	11 (45.8%)	6 (54.5%)	0.725^a^
ICU stay (d), median (IQR)	13 (6, 17)	15 (9.5, 19.5)	10 (6, 14)	0.119^b^
Hospital stay (d), median (IQR)	17 (10, 26)	18 (13.5, 26)	11 (9, 29)	0.201^b^
Survivors (*n*, %)	27 (77.1%)	19 (79.2%)	8 (72.7%)	0.685^a^

^a^ Fisher's exact test.

^b^ Mann–Whitney rank sum.

ALSS, artificial liver support system; PRISM III, pediatric risk of mortality III; HE, hepatic encephalopathy; PE, plasma exchange; DPMAS, double plasma molecular adsorption system; CRRT, continuous renal replacement therapy; AKI, acute kidney injury; MODS, multiple organ dysfunction syndrome.

**Figure 5:**
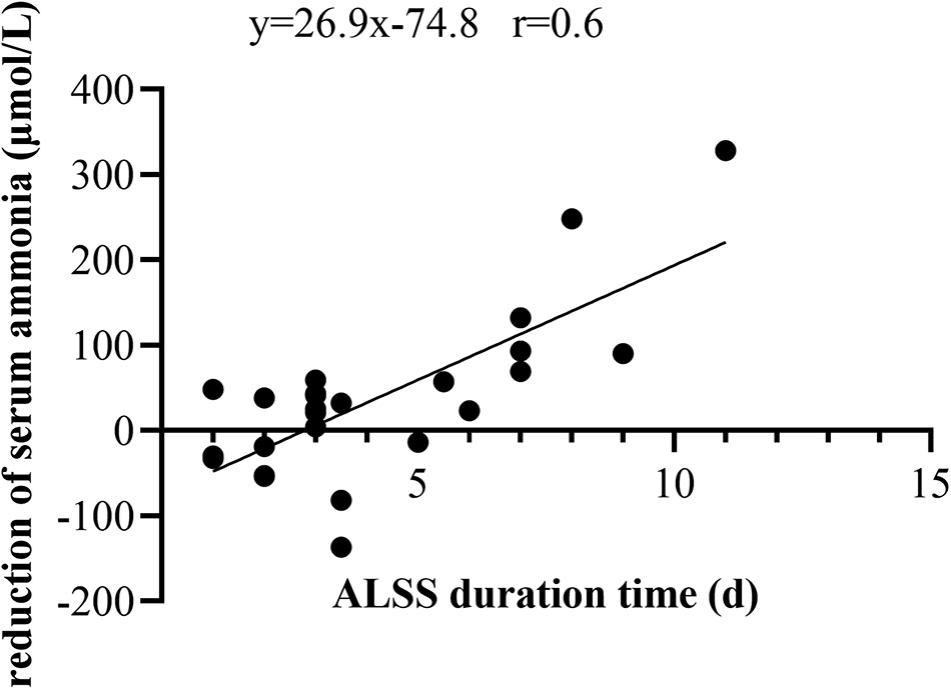
Correlation and linear regression between ALSS duration and reduction in serum ammonia.

Following the same procedure, thirteen patients were excluded due to incomplete data, leaving twenty-six patients to examine factors influencing serum ammonia reduction after ALSS. Among the multiple variables compared between the serum ammonia-responsive group (ammonia _before_ ALSS > ammonia _after ALSS_, *n* = 18) and the serum ammonia-unresponsive group (ammonia _before_ ALSS < ammonia _after ALSS_, *n* = 8), a longer ALSS duration was significantly related to ammonia reduction (Table 3). A positive correlation was also observed between ALSS duration and serum ammonia reductions after ALSS (*r* = 0.6 by Spearman rank correlation, *P* < 0.001, Figure 5).

**Table 3:** Univariate analysis between the serum ammonia-responsive group and the unresponsive group.

	All (*n* = 26)	Ammonia-responsive group (*n* = 18)	Ammonia-unresponsive group (*n* = 8)	*P* value
Age (year), median (IQR)	8 (1.5, 10)	7.5 (1, 10)	8 (6, 12)	0.253^b^
Male (*n*, %)	11 (42.3%)	7 (38.9%)	4 (50%)	0.683^a^
PRISM III, median (IQR)	13 (7, 17)	13 (7, 17)	13 (7, 17)	0.911^b^
HE at admission (*n*, %)	11 (42.3%)	10 (55.6%)	1 (12.5%)	0.084^a^
Onset to PICU (d), median (IQR)	6.5 (3, 9)	5.5 (3, 8)	8 (3.5, 10)	0.52^b^
Etiology (*n*, %)				
Infection/toxication	9 (34.6%)	7 (38.9%)	2 (25%)	0.667^a^
Other etiology	17 (65.3%)	11 (61.1%)	6 (75%)	
ALSS start time (h), median (IQR)	26 (21, 38)	24 (17, 38)	27 (23, 44)	0.277^b^
ALSS duration time (d), median (IQR)	2 (2, 4)	3 (3, 7)	2 (1, 3.5)	0.027^b^
ALSS modalities (*n*, %)				
PE only	6 (23%)	6 (33.3%)	0	0.169^a^
CRRT only	1 (3.8%)	0	1 (12.5%)	
PE + CRRT	12 (46.1%)	7 (38.9%)	5 (62.5%)	
PE + DPMAS	3 (11.5%)	2 (11.1%)	1 (12.5%)	
PE + DPMAS + CRRT	4 (15.3%)	3 (16.7%)	1 (12.5%)	
Complications				
HE (*n*, %)	20 (76.9%)	15 (83.3%)	5 (62.5%)	0.33^a^
AKI (*n*, %)	2 (7.7%)	0	2 (25%)	0.086^a^
MODS no. ≥ 3 (*n*, %)	14 (53.8%)	8 (44.4%)	6 (75%)	0.216^a^
Ventilation (*n*, %)	13 (50%)	8 (44.4%)	5 (62.5%)	0.673^a^
Ventilation duration (d), median (IQR)	3 (2.5, 6)	3.5 (2, 7)	3 (2, 8.5)	0.823^b^
Vasoactive agents (*n*, %)	13 (50%)	9 (50%)	4 (50%)	1^a^
ICU stay (d), median (IQR)	14.5 (8, 18)	12.5 (7, 18)	16 (11.5, 23)	0.243^b^
Hospital stay (d), median (IQR)	17 (10, 25)	16 (10, 21)	22 (12.5, 33)	0.07^b^
Survivors (*n*, %)	20 (76.9%)	14 (77.8%)	6 (75%)	1^a^

^a^ Fisher's exact test.

^b^ Mann−Whitney rank sum.

ALSS, artificial liver support system; PRISM III, pediatric risk of mortality III; HE, hepatic encephalopathy; PE, plasma exchange; DPMAS, double plasma molecular adsorption system; CRRT, continuous renal replacement therapy; AKI, acute kidney injury; MODS, multiple organ dysfunction syndrome.

## Discussion

PALF is a rare but fatal disease with mortality varying from 24%–75% (8, 12). Standard medical therapies have been demonstrated to be inadequate. Liver transplantation is the ultimate treatment, but spontaneous recovery with a native liver is the ideal outcome, which avoids complications and mortality (9, 13). Thus, ALSS is becoming a promising technique. However, the effect of ALSS in PALF is lacking and controversial. Using the cohort of patients with PALF, we demonstrate the biochemical efficiency of ALSS. In addition, the reduction of TBIL after ALSS is dependent on etiology, and a longer ALSS duration time is related to ammonia reduction. To the best of our knowledge, this was the first study to explore risk factors for ALSS efficiency on TBIL and serum ammonia levels in PALF.

ALSS use in patients with acute liver failure has increased greatly recently, especially PE utilization. We mainly used PE-based hybrid extracorporeal therapies. PE has a beneficial effect by delivering biologically active substances contained in fresh frozen plasma and removing toxic factors, cytokines, and vasoactive substances (14). This is the most important mechanism by which PE can improve liver function. However, when PALF is complicated by AKI, MODS, fluid overload, hyperammonemia and other complications, PE alone is not sufficient. Therefore, PE combined with CRRT, which will help optimize fluid balance and electrolytes and remove water-soluble small-molecule toxins, including ammonia, urea and creatinine, must be considered. CRRT can also enhance the removal of inflammatory mediators (9, 13). DPMAS helps remove toxic metabolites, including bilirubin and inflammatory mediators, especially when PE is not available (15). No definitive criteria for the initiation or discontinuation of PE, DPMAS or CRRT in PALF patients have been established (16). Selection of a suitable ALSS modality at the right time is crucial to evaluate the efficacy of ALSS for patients with PALF. In our study, although no differences in ALSS patterns were observed between groups, we still believe that the hybrid ALSS pattern may be more reasonable based on the complex condition of PALF (9).

We found that ALSS use was associated with a trend toward reduced median serum ALT, TBIL, INR, and ammonia levels. These findings are consistent with previous reports. Various studies have shown that PE can significantly improve biochemical parameters and transplant-free survival in adults with acute liver failure (5, 17, 18). In 2018, a multicenter cohort study revealed that CRRT was associated with a reduction in serum ammonia levels and improvement in 21-day transplant-free survival (3). In the few available studies of PE-based ALSS focusing on pediatric patients, the authors also reported improvements in biochemical parameters (12, 19). Arikan's report suggested that hybrid extracorporeal therapies can be effectively implemented in PALF as a bridge to transplantation (9). Although we did not have a control group to confirm that the effect of ALSS was better than that of standard medical treatment, we found a relatively low mortality rate compared to those in the published literature (23.1% vs. 24%–75%) (8, 12).

PALF is caused by multiple factors, such as infection, metabolic diseases, immunity and toxicity. Although extensive investigations have been performed, indeterminate PALF still occurs in up to 50% of cases (11). In the present study, we found that the most common cause of PALF was indeterminate, which was consistent with the findings of previous worldwide studies (16). We found that infectious and toxic causes were associated with poor TBIL reduction. This finding may be partly because the “acute insult” progresses more quickly and results in the development MODS or encephalopathy more rapidly than relatively chronic causes, such as metabolic diseases. It did not allow ALSS to work better. This finding was consistent with previous reports suggesting that “pure” PALF, including toxicity and infection, was associated with worse outcomes in children (20, 21). Several pediatric studies have focused on the impact of etiology on prognosis (11). For instance, indeterminate PALF and Wilson disease were associated with a low likelihood of spontaneous recovery. In this study, these two etiologies (i.e., the other etiologies in this study) were associated with the effective reduction of TBIL after ALSS, suggesting that ALSS can serve as a bridge for liver transplantation in these two etiologies. We also found that a longer ALSS duration was associated with blood ammonia reduction. The primary reason for this could be the key role of CRRT in serum ammonia removal, and the ALSS duration is almost the same as the CRRT duration in this study.

Based on previously published literature, younger age, the presence of advanced HE, the need for ventilator support or vasopressors, and MODS were related to worse outcomes (22). Therefore, these variables have been hypothesized to be related to ALSS failure. However, in this study, we did not identify any correlation between these variables and ALSS treatment outcomes. This lack of significance may be due to the small sample size.

Although ALSS may positively impact PALF patients' clinical outcomes, this method represents only one therapeutic strategy to partially compensate for liver function. The timely treatment of multiple organ failures in the PICU is also very important and has contributed to the overall improvement of these patients' clinical outcomes.

This study has some limitations that must be acknowledged. First, this was a retrospective cohort and therefore may have been prone to information bias and selection bias. The etiology was identified based on clinical conditions at the time, so the variation in etiological recognition existed across time. There is also a lack of standardization with regard to ALSS, and its treatment plans were individualized by intensive care physicians based on the clinical status at the time and local protocols. Second, this study involved a small sample size and was performed at a single center. The study may have been underpowered to detect some risk factors, so we could not perform a multifactor analysis. However, this is a common problem in single-center retrospective observational studies involving the etiology of PALF. Although prospective multicenter studies are necessary, this research will take a long time and require significant effort due to the relatively low prevalence of PALF. We hope that our retrospective studies will provide certain clues for future prospective multicenter studies. Above all, despite these limitations, our study is significant in contributing to the limited reports of PALF treated with ALSS.

## Conclusion

Our experience suggests that ALSS can effectively reduce serum ALT, TBIL, INR and ammonia levels and may reduce mortality. The reduction in TBIL after ALSS is dependent on etiology. A longer ALSS duration was associated with serum ammonia reduction. Further prospective multicenter studies are needed to examine the efficacy of ALSS on mortality and characterize the factors associated with ALSS treatment failure with the ultimate goal of predicting treatment outcomes and improving the prognosis of children with PALF.

## Data Availability

The raw data supporting the conclusions of this article will be made available by the authors, without undue reservation.
